# A Type A and Type D Combined Personality Typology in Essential Hypertension and Acute Coronary Syndrome Patients: Associations with Demographic, Psychological, Clinical, and Lifestyle Indicators

**DOI:** 10.1371/journal.pone.0161840

**Published:** 2016-09-02

**Authors:** Patrizia Steca, Marco D’Addario, Maria Elena Magrin, Massimo Miglioretti, Dario Monzani, Luca Pancani, Marcello Sarini, Marta Scrignaro, Luca Vecchio, Francesco Fattirolli, Cristina Giannattasio, Francesca Cesana, Salvatore Pio Riccobono, Andrea Greco

**Affiliations:** 1 Department of Psychology, University of Milan “Bicocca”, Milan, Italy; 2 Department of Medical and Surgical Critical Care, Cardiac Rehabilitation Unit, University of Florence and Azienda Ospedaliero-Universitaria Careggi, Florence, Italy; 3 Health Science Department, University of Milan “Bicocca”, Milan, Italy; 4 Cardiology IV, Cardiovascular “A.De Gasperis” Department, Niguarda Ca’ Granda Hospital, Milan, Italy; IRCCS Istituto Auxologico Italiano, ITALY

## Abstract

Many studies have focused on Type A and Type D personality types in the context of cardiovascular diseases (CVDs), but nothing is known about how these personality types combine to create new profiles. The present study aimed to develop a typology of Type A and Type D personality in two groups of patients affected by and at risk for coronary disease. The study involved 711 patients: 51.6% with acute coronary syndrome, 48.4% with essential hypertension (mean age = 56.4 years; SD = 9.7 years; 70.7% men). Cluster analysis was applied. External variables, such as socio-demographic, psychological, lifestyle, and clinical parameters, were assessed. Six groups, each with its own unique combined personality profile scores, were identified: Type D, Type A-Negatively Affected, Not Type A-Negatively Affected, Socially Inhibited-Positively Affected, Not Socially Inhibited, and Not Type A-Not Type D. The Type A-Negatively Affected cluster and, to a lesser extent, the Type D cluster, displayed the worst profile: namely higher total cardiovascular risk index, physical inactivity, higher anxiety and depression, and lower self-esteem, optimism, and health status. Identifying combined personality profiles is important in clinical research and practice in cardiovascular diseases. Practical implications are discussed.

## Introduction

The appearance and clinical progression of cardiovascular diseases (CVDs) are associated with a range of psychosocial variables, including personality factors. Since the 1960s, many studies have focused on personality, and more specifically, on personality types: Type A [1, 2] and Type D [3, 4] are the personality profiles most often studied in the context of CVDs.

The Type A personality, also known as the Type A behaviour pattern, dominated research on the links between personality factors and cardiac diseases during the 1970s and 1980s. It is characterized by high competition, ambition, motivation to achieve, impatience, aggressiveness, social hostility, and vulnerability to stress. In the prospective Western Collaborative Group Study [5, 6], a unique (i.e., independent of other risk factors) association between Type A and coronary heart disease (CHD) was observed during an 8.5-year follow-up. Various studies have supported these findings in both other CHD populations and in people with other CVD pathologies [7, 8]. Several contributions have examined and replicated the negative effect of Type A personality on essential hypertension (HYP), defined as a chronic elevation of systolic and diastolic blood pressure, which constitutes a significant risk factor for coronary and cerebral vascular diseases [9–13]. Moreover, the detrimental role of a Type A personality has also been identified in patients after an acute coronary syndrome (ACS) diagnosis; it was found to independently predict sudden cardiac death in patients with at least one documented myocardial infarction [14]. However, many other studies have obtained contrasting results in relation to CHD [15–17], HYP [18–20], and patients after ACS [21–23]. These discouraging findings have led researchers to shift their focus from the Type A personality towards its specific components; hostility and “anger-in” factors have been suggested as the toxic components of Type A personality that play a particularly significant role in CVDs [24–30]. Hostility refers to a stable predisposition to experience a combination of anger, irritation, annoyance, and resentment. Anger-in has been conceptualized as the tendency to hold back expressions anger against others, even if such expression might be appropriate [31]. Various studies have found that hostility and anger-in successfully predicted the incidence of a variety of manifestations of CVD, including CHD and HYP [7, 24, 26, 28, 31–33). Although many subsequent studies, reviews and meta-analyses concluded that Type A personality was not a valid indicator of cardiac prognosis or hard endpoints in CVDs [29, 34–38], some researchers have suggested that this personality pattern may suffered a premature demise. Recent research has shown that interventions for Type A personality improved depression in CHD patients [39–40]. Furthermore, other studies highlighted that intervention programs targeting, among others, Type A personality subcomponents, such as time urgency, impatience, irritation, and hostility, decrease the risk of recurrent CVDs and recurrent acute myocardial infarction [41] and cardiac endpoints for female CHD patients [42]. Moreover, there is continued research interest in the relationship between Type A personality and different outcomes, such as the presence of CHD, lifestyle, and quality of life in people with CVD and other pathologies [10, 43–47].

More recently, a new personality type, defined as the Type D or distressed personality, has been associated with CVD [3, 4, 48, 49]. This personality type is defined by two dimensions: negative affectivity and social inhibition traits. Hence, it is characterized by social anxiety and the tendency to experience negative emotions and affectivity over time and situations and to inhibit self-expression in social interactions, with difficulties in managing interpersonal relationships. The prevalence of Type D varies between 13% and 25% in the general population and between 26% and 53% in CVD patients [48, 50–53]. Furthermore, a higher prevalence of Type D has been found among HYP patients compared with healthy controls [52]. Type D personality has been proposed as a prognostic factor for mortality in CVD independent of other biological risk factors and disease severity [3]. Moreover, Type D has been shown to predict adverse outcomes, including mortality, morbidity, and impaired health status, in several groups of patients suffering from different types of CVD [54], CHD [4, 55], and ACS [56]; similar results have been found in patients with ischaemic heart disease after percutaneous coronary intervention [53] with coronary artery bypass grafting [57] and in patients with peripheral arterial disease [50]. Moreover, Type D personality has been associated with lower treatment adherence [58] and emotional distress in CHD patients [56, 59–62]. Although many studies have found support for the negative role of the Type D personality, the prognostic effects of Type D decreased considerably over time. Recent studies with large samples have accumulated consistently null findings. Moreover, recent systematic reviews and meta-analyses have suggested that earlier Type D studies overestimated the personality type’s prognostic relevance, neglecting the problems related to small sample sizes [63–65]. Furthermore, other issues have been raised concerning the construction of the Type D personality in terms of research design, methodology and statistical aspects: for example, the use of median-split or dichotomized continuous measures on arbitrarily chosen cut-points; the use of cross tabs to select the patients in the high quadrant for negative affectivity and social inhibition levels and compared them to the patients in the other three quadrants; when regression models were used to test multivariate predictions, an abuse of selection by including possible covariates-confounders (and excluding others) depending on the impact of negative affectivity and social inhibition variables; and the flexible extension or contraction of follow-up periods, depending whether Type D personality significantly predicted mortality or other outcomes. All of these aspects could magnify the results related to Type D personality [66].

Almost all previous research on personality types was interested in Type A or Type D personalities separately. To our knowledge, only one correlational (German) study has investigated the two patterns of personality together, both in CHD patients and in a healthy sample. The results showed that negative affectivity and social inhibition were correlated with hostility, anger, cynicism, and physical aggression in both populations. Moreover, the participants classified as Type D reported higher levels of anger, cynicism, and hostility than the participants classified as non-Type D [67]. However, nothing is known about how these personality types integrate to form different profiles. Therefore, according to the person-centred approach [68], the present study examined Type A and Type D combined personality profiles in two groups of patients affected by and at risk for CVD. As stated by Magnusson [68], this approach allows for the study of individual functioning through an integrative view of the person that cannot be completely understood with traditional variable-centred approaches that consider individual components of the person in isolation. Magnusson [69] have stressed the importance of considering the global personality configurations instead of a single variable, since, for example, the correlations that could exist in the population between two characteristics may reflect the influence of a small group of subjects characterized by a peculiar profile. The author highlighted that the consideration of multiple variables and the relations between the derived constellations of variables may explain and predict future behaviours better than exploring the role of just one single variable. We hypothesized that the combination of Type A and Type D personality patterns may have more clinically meaningful implications than the two personality types separately.

### Aims of the study

We studied how Type A and Type D personality factors could integrate or combine into new profiles using the cluster analysis technique. Furthermore, we examined how the resulting profiles or types were related to external variables, such as socio-demographic, psychological, lifestyle, and clinical indicators.

Cluster analysis is a multivariate method specifically developed to classify a sample of participants according to set of measured variables into a number of different groups, so that similar individuals are placed in the same group [70]. This study is based on continuous-level variables related to Type A and Type D personality, contrary to the procedures applied in previous research in this field that has used categorical conceptions based on arbitrarily chosen cut-off scores. Consequently, the present study aimed to identify an empirically derived integrated and combined classification or typology of Type A and Type D personality configurations in two groups of patients affected by and at risk for CVD: those with ACS, the most common type of CVD, and those with essential HYP, the main risk factor for CVD. The resulting clusters or groups were examined for differences in socio-demographic characteristics (i.e., age, gender, partner relationship status, occupation, education, and number of friends), psychological variables (i.e., anxiety, depression, self-esteem, optimism, illness perception, and self-rated health), lifestyle (i.e., diet, physical activity, alcohol consumption, and cigarette smoking behaviour), and clinical parameters (i.e., total cardiovascular risk index).

These external variables were selected based on theoretical reasons cited in the literature that highlights the link between these factors and CVD. The clinical evolution and progression of established CVD are related to a range of psychosocial factors, such as anxiety and depression, which may partially explain the progression and recurrence of these diseases [71–74]. In addition to this focus on negative factors, other studies have underlined the role of psychological variables that may act as protective factors that buffer the effects of CVD and progression. Recent studies have demonstrated the role of resiliency factors, such as optimism, self-esteem, and positive illness perception, in the process of adjusting to CVD [75–80]. Similarly, health and illness representations have been shown to have prognostic value in predicting adverse CVD clinical events [81–84]. Moreover, the literature suggests that people with poor social ties or a small social network are at an increased risk of CVD [85–87]. Furthermore, the importance of a healthy lifestyle (i.e., the cessation of cigarette smoking, high consumption of vegetables and fruits, a low-fat diet, moderate alcohol intake, and physical exercise) and controlling modifiable risk factors (obesity, diabetes mellitus, and dyslipidaemia) are crucial aspects for the prevention and treatment of CVD, as emphasized in international guidelines on prevention and management of CVD and HYP [88–90].

Because cluster analysis is a data-driven procedure, any number of clusters could be identified as the most optimal. Due to a lack of prior research on a combined typology of Type A and Type D personality, we did not have clear expectations regarding the number of clusters that would emerge. However, based on previously identified personality typologies, we expected at least one cluster marked by high levels of Type A personality and one group characterized by Type D personality. Moreover, we hypothesized that there would be one or more clusters marked by the presence of both Type A and Type D characteristics. Finally, we expected to find clusters characterized by low levels of Type A and D components that could be differentiated by their profile of external correlates. More specifically, we expected that the groups characterized by low levels of Type A and D components would show the most optimal psychological, lifestyle, and clinical profiles. In line with previous studies demonstrating that people with low levels of Type A or Type D characteristics reported lower psycho-emotional distress and higher levels of health and health-related behaviours than Type A or Type D individuals [54, 58, 91–92], we hypothesized less psycho-emotional distress, more social connectedness, a positive orientation, better health and illness representations, better dietary habits, more physical activity, reduced alcohol consumption and cigarette smoking, and a lower presence of CVD risk factors in groups with low levels of Type A and D characteristics. At the same time, we hypothesized a less positive profile in terms of these external correlates for the clusters marked by high levels of Type A personality and/or Type D personality. In fact, in relation to Type A personality, previous studies have reported a worse lifestyle, more physical symptoms, more negative clinical indices, and a worse perception of health [93–95]. Regarding Type D, previous findings have demonstrated that it was associated with higher levels of anxiety, depression, and negative illness perceptions; moreover, Type D personality was associated with poor health-related quality of life and unhealthy behaviours [48, 58, 96–101]. Considering these previous results together, we expected that the cluster or clusters marked by the presence of Type A and Type D characteristics together would show the worst profile in terms of psychological, lifestyle, and clinical indicators.

## Materials and Methods

### Participants and procedure

From February 2011 to May 2014, we recruited consecutive patients with essential arterial HYP and those newly diagnosed with ACS who were referred to different hospitals in northern Italy. Eligible patients received written information about the study and a consent form to be signed. Patients who were eligible to participate in the study were between 30 and 80 years of age, had essential arterial HYP (i.e., they were already receiving pharmacological treatment and/or had elevated blood pressure values, including systolic blood pressure (SBP) > = 140 mmHg and/or diastolic blood pressure (DBP) > = 90 mmHg) or were newly diagnosed with ACS (even if they had been treated with coronary angioplasty and stenting) and had sufficient Italian language skills. Patients with cognitive deficits or other major pathologies (such as cancer) were excluded. Although a structured interview is considered a better approach than self-report questionnaires to assess psychological factors (e.g., Type A personality, anxiety, and depression) in the CDV context, we collected data using self-report questionnaires administered to the participants by a trained researcher. In fact, given the health state of the ACS patients involved in the study, we administered brief questionnaires to measure all of the variables considered in this study and aimed to engage the patient for no more than 30 minutes. The patients with HYP were asked to complete the assessment questionnaire during a medical examination at the care centre; the ACS patients were asked to complete the questionnaire during rehabilitation 2 to 8 weeks after hospitalization. For all of the patients, the physicians collected a set of clinical data related to risk factors for CVD. The study was approved by the Ethical Committee of the University of Milan-Bicocca.

We asked 774 participants with HYP or ACS to participate in the study, 63 of whom refused for personal reasons (e.g., no interest in participating). The non-participating patients did not differ significantly from the respondents with respect to age or gender. The study sample included 711 patients (response rate = 91.9%), representing a sufficiently large sample for conducting cluster analysis [102]. Nearly half of the sample (48.4%) comprised patients with HYP. The participants were primarily male (70.7%) and married (75.1%), with a mean age of 56.4 years (*SD* = 9.7); 42.2% of patients had graduated from secondary school, and the majority were still working (55.1%). Regarding clinical data related to risk factors for CVD, approximately half of the sample (49.6%) had a family history of CVD, 78.1% presented dyslipidaemia, 46.4% presented obesity, 49.2% presented abdominal obesity, 26.9% had diabetes, and 53.6% were smokers. A summary of the participants’ characteristics is presented in Table 1.

**Table 1:** Patients’ characteristics.

Patients’ Characteristics			
Sociodemographic			
Age, mean ± SD	56.4 ± 9.7	Employment status	N (%)
Gender	N (%)	• Employed	390 (55.1)
• Male	503 (70.7)	• Retired	194 (27.4)
• Female	208 (29.3)	• Unemployed	45 (6.4)
Educational level	N (%)	• Homemaker	34 (4.8)
• No title	4 (0.6)	• Retired with some work activities	45 (6.4)
• Primary school	78 (11.0)	Marital Status	N (%)
• Middle school	219 (30.9)	• Single	87 (12.2)
• High school	299 (42.2)	• Married	534 (75.1)
• Graduate school	90 (12.7)	• Divorced/Separated	70 (9.8)
• Postgraduate school	19 (2.7)	• Widowed	20 (2.8)
**Clinical**			
Pathology	N (%)	Risk factors	N (%)
• HYP	344 (48.4)	• Family History	353 (49.6)
• ACS	367 (51.6)	• Smoking History	381 (53.6)
		• Obesity	330 (46.4)
Risk factors, mean ± SD	4.5 ± 2.0	• Abdominal obesity	350 (49.2)
		• Dyslipidemia	555 (78.1)
		• Diabetes	191 (26.9)
		• SBP> = 140 and/or DBP> = 90 mmHg	356 (50.1)

Note: HYP = hypertension, ACS = acute coronary syndrome, SBP = systolic blood pressure, DBP = diastolic blood pressure

### Variables and instruments

#### Psychological variables—Type A personality

We used items originating from the Cognitive Behavioural Assessment Form Hospital battery (CBA-H) [103, 104] and newly researcher-constructed items to measure the personality characteristics of the Type A behaviour pattern. People who score high on the Type A scale are described as hard-driving, fast moving, work-oriented individuals who frequently become impatient, irritable, and annoyed. We used 12 items to evaluate competitiveness (2 items), impulsivity (2 items), job involvement (2 items), leadership (3 items), and hostile attitudes (3 items). An example item is “You don’t get anything in life without being competitive”. Each item was rated on a 5-point scale, ranging from “Absolutely false for me” (1) to “Absolutely true for me” (5).

Because we used an extended version of this scale, we performed Exploratory Factor Analysis (EFA) and Confirmatory Factor Analysis (CFA) with Mplus software—Version 6.11 [105] to test the validity of the scale. We performed an EFA on approximately half of the participants (selected randomly) to evaluate the factor solution with 5 latent factors using a principal axis factoring extraction method (a method based on the variables’ communality) and a promax rotation because the factors were expected to be correlated. The pattern of factor loadings showed that no item displayed a loading lower than .32, the cut-off for substantial loading [106], and the results indicated that there were no cross-loadings. Furthermore, CFA of the second half of the participants was used to check the factor structure of the scale. Hu and Bentler’s guidelines [107] for various fit indices were used to determine whether the expected model was plausible based on the data. The first index used was the chi-square test statistic; considering the sensitivity of the chi-square statistic to the sample size, other goodness-of-fit indices were used: the comparative fit index (CFI ≥ .90 indicates an adequate fit), the root-mean square error of approximation (RMSEA ≤ .08 indicates a good fit), and the standardized root-mean-square residual (SRMR ≤ .08 indicates an adequate fit). The CFA indicated that the five-factor model fit the data adequately, χ^2^ = 71, df = 49, *p* < .01, CFI = .93, RMSEA = .05, SRMR = .04.

Furthermore, because when too many variables are used in cluster analysis, it is difficult to meaningfully describe the clusters [108], we performed a second-order EFA (principal axis factoring and promax rotation) to reduce the dimensionality of this scale. Only one factor attained Kaiser's criterion of an eigenvalue above 1; the pattern of factor loadings showed that no first-order factor displayed a loading lower than .32. Moreover, a second-order CFA indicated that the one-factor model fit the data adequately, χ^2^ = 80, df = 49, *p* < .01, CFI = .92, RMSEA = .05, SRMR = .04. As in previous research [43, 109, 110], these results indicated that competitiveness, impulsivity, job involvement, leadership, and hostile attitudes could be reduced to a single factor, labelled Type A personality. For this scale, the scores were calculated as the mean item scores, where higher scores indicate the greater presence of a Type A behaviour pattern. In line with recommendations by Cronbach [111], the scale showed an adequate internal consistency (Cronbach’s alpha was .71).

#### Type D personality

We used items originating from the Type D 14-item Scale, DS14 [112, 113] and researcher-constructed items to measure the characteristics of the type D personality. The dimensions of negative affectivity and social inhibition were measured by 4 items each. All of the items were rated on a 5-point Likert scale ranging from “Absolutely false for me” (1) to “Absolutely true for me” (5). An example item is “I often find myself worrying about something”.

Similar to the Type A personality scale, we performed EFA and CFA. We performed an EFA on approximately half of the participants (selected randomly) to evaluate the factor solution with 2 latent factors using a principal axis factoring extraction method and a promax rotation. The pattern of factor loadings showed that no item displayed a loading lower than .32 and that there were no cross-loadings. Furthermore, the CFA of the other half of the selected participants was used to check the model’s factor structure. The results of this analysis indicated that the two-factor model fit the data adequately, χ^2^ = 25, df = 19, *p* = n.s., CFI = .99, RMSEA = .03, SRMR = .03.

We performed a second-order EFA to reduce the dimensionality of this scale, but this analysis performed poorly, suggesting that Type D personality could not be reduced to fewer than two latent variables. For these factors, the scores were calculated as the mean item scores, where higher scores indicated greater negative affectivity and social inhibition. The scales showed adequate internal consistency (Cronbach’s alpha was .80 for negative affectivity and .73 for social inhibition).

#### Demographic and social connectedness indicators

The participants were asked to report their socio-demographic information, including gender, age, marital status, employment status, and educational level. Moreover, to gather information about social connectedness, the patients were asked to report their number of friends by answering the question “Among the people you know, how many do you consider your FRIENDS?” on a 4-point Likert scale (1 = “none”, 2 = “1–5 friends”, 3 = “6–10 friends”, 4 = “more than 10 friends”).

#### Psycho-emotional distress—anxiety and depression

We used the Italian version of the Hospital Anxiety and Depression Scale (HADS), a 14-item self-report measure developed to screen for emotional distress in medical patients [113, 114]. The HADS has been shown to be a reliable and well-validated scale in various studies of patients with CVD [115, 116]. The participants reported their feelings and moods on a 4-point Likert scale; an example item is “I’ve lost interest in caring for my physical appearance”, and the possible answers are 1 = “completely”, 2 = “I don’t care for it as much as I should”, 3 = “I care for it a bit less than I should”, 4 = “I don’t care for it like before”. Summed scores are calculated separately for anxiety and depressive symptoms; the total score ranges from 0 to 21, and higher scores indicate a greater presence of mood disorders. The CFA indicated that the two-factor model fit the data adequately, χ^2^ = 252, df = 76, p < .001, CFI = .93, RMSEA = .06, SRMR = .04. The scales showed an adequate internal consistency (Cronbach’s alpha was .81 for anxiety and .73 for depression).

#### Positive orientation—self-esteem and optimism

We used reduced Italian versions of the Rosenberg Self-Esteem Scale [117] and of the Life Orientation Test Revised [118–120]. Self-esteem was calculated as the mean of the responses to 3 items on which the participants indicated the extent to which they felt they possessed good qualities, accepted their own characteristics, and had achieved personal success or experienced failure. Each item was rated on a 4-point scale (from 1 = “strongly disagree” to 4 = “strongly agree”); a sample item is ‘‘I am able to do things as well as most other people”. Optimism was calculated as the mean of the responses to 3 items; the respondents indicated the extent to which they agreed or disagreed with each item on a 5-point Likert scale that ranged from 1 = “strongly disagree” to 5 = “strongly agree”. A sample item is “I’m always optimistic about my future”. The CFA indicated that the two-factor model fit the data adequately, χ^2^ = 47, df = 8, p < .001, CFI = .95, RMSEA = .08, SRMR = .05. The scale showed adequate internal consistency (Cronbach’s alpha was .68 for self-esteem and .60 for optimism).

#### Health and illness representations—illness perception

The Italian version of the Brief Illness Perception Questionnaire (Brief-IPQ) [121, 122] was used to measure different components of the patients’ illness perception, as defined by Leventhal et al. [123]. The Brief-IPQ has 8 items that assess the following illness perceptions: (1) consequences, referring to the patient’s perception of the impact of the illness on his/her life; (2) timeline, reflecting the individual’s feelings about how long the illness will last; (3) personal control, referring to the patient’s perception of his/her own degree of control over the illness; (4) treatment control, reflecting the perceived usefulness of the treatment; (5) intensity, corresponding to the perceived intensity of the illness symptoms; (6) concern, referring to how much the patient worries about the illness; (7) emotions, reflecting the extent to which the illness affects the patient’s emotions; and (8) comprehensibility, corresponding to the extent to which the patient thinks he/she understands his/her illness. All of the items were rated using a 5-point Likert scale. The Brief-IPQ contains an open-response item that assesses patients’ causal attributions of the illness; this item was not included in the present study because it does not add information to the overall score. Following a procedure adopted through previous research, the overall score was calculated as the sum of the eight items’ scores [80, 124, 125]. The total scores reflect the overall negativity of the patients’ illness perception. A CFA indicated that the one-factor model fit the data adequately, χ^2^ = 106, df = 20, p < .001, CFI = .91, RMSEA = .08, SRMR = .06. The summary score had an adequate internal consistency (Cronbach’s alpha was .61).

#### Self-rated health

The patients were asked to rate their general state of health as follows: 1 = “excellent”, 2 = “very good”, 3 = “good”, 4 = “fair”, or 5 = “poor”.

#### Lifestyle—diet

To measure dietary habits, an Italian version of the Mediterranean Diet Scale [126] was used. Following a procedure used in previous research [127], the frequency of consumption of both beneficial foods (vegetables, fruit, whole grains, fish, legumes, olive oil) and detrimental foods (wine, butter and margarine, red or processed meat) was assessed using a dichotomous variable (1 = “healthy consumption” and 0 = “unhealthy consumption”) for all foods except fats, which were considered healthy (score of 1) if the consumption of olive oil was higher than that of butter/margarine and unhealthy (score of 0) otherwise. The responses were classified into four categories: 0 = “inadequate”, 1 = “partial”, 2 = “good” and 3 = “excellent”.

#### Physical activity

Physical activity was measured using the Rapid Assessment of Physical Activity Questionnaire [128]. This questionnaire included 9 yes/no items that assessed the type and amount of reported physical activity. Based on their responses to the items, the participants were categorized as inactive (coded as “0”), or meeting the target for healthy physical activity (coded as “1”; i.e., 30 minutes of moderately intense physical activity 5 or more days per week/20 minutes of vigorously intense physical activity 3 or more days per week).

#### Alcohol consumption

The total alcohol intake of each participant was computed as the mean consumption of beer (1 = “I don’t drink beer”, 2 = “Up to 2 glasses a day”, 3 = “3 or 4 glasses a day”, 4 = “More than 4 glasses a day”), wine (1 = “I don’t drink wine”, 2 = “Up to 2 glasses a day”, 3 = “3 or 4 glasses a day”, 4 = “More than 4 glasses a day”), and spirits (1 = “I don’t drink spirits”, 2 = “1 glass occasionally—a few times a year”, 3 = “1 glass habitually—ex. every week, always after meals, etc.”, 4 = “More than 1 glass habitually—ex. every week, always after meals, etc.”), as used in previous research [129].

#### Cigarette smoking behaviour

One item was used to measure the participants’ smoking behaviour: “How many cigarettes do you smoke a day?”. The scale ratings were 0 = “non-smoker”, 1 = “10 or fewer cigarettes a day”, 2 = “11–20 cigarettes a day”, 3 = “21–30 cigarettes a day”, 4 = “31 or more cigarettes a day”, as used in previous research [130].

#### Clinical evaluation

The physicians collected data regarding different risk factors for CVD, including sex, age, smoking behaviour, obesity, abdominal obesity, diabetes mellitus, dyslipidaemia, elevated blood pressure, and a family history of premature CVD. The relationship between the onset of CVD and the concomitant presence of multiple interacting CVD risk factors has been certified in previous studies [88]. Moreover, reductions in CVD risk factors can account for more than half of the decrease in CHD deaths [131]. Among HYP patients, too, the control of concomitant CVD risk factors has resulted in a reduction of CVD morbidity and mortality [132].

A “Total Cardiovascular Risk Index” (TCRi) was calculated for each patient based on the sum of CVD risk factors, with 1 point assigned for each risk factor present. For the HYP patients, the “European Guidelines for the Management of Arterial Hypertension” were used [89], and the following were considered risk factors: male sex, age (men > = 55 years; women > = 65 years), smoking, obesity (BMI > = 30 kg/m^2^ [height^2^]), abdominal obesity (waist circumference: men > = 102 cm, women > = 88 cm), diabetes mellitus, dyslipidaemia (total cholesterol > 190 mg/dL and/or low-density lipoprotein cholesterol > 115 mg/dL and/or high-density lipoprotein cholesterol: men < 40 mg/dL, women < 46 mg/dL and/or triglycerides > 150 mg/dL), elevated blood pressure values (SBP > = 140 mmHg and/or DBP > = 90 mmHg), and a family history of premature CVD (men aged < 55 years; women aged < 65 years). For the ACS patients, the following risk factors were selected based robust scientific evidence from various studies and guidelines [90, 133, 134]: male sex, age (men > = 55 years; women > = 65 years), smoking, obesity (BMI > = 25 kg/m^2^ [height^2^]), abdominal obesity (waist circumference: men > = 94 cm, women > = 80 cm), diabetes mellitus, dyslipidaemia (total cholesterol > 155 mg/dL and/or low-density lipoprotein cholesterol > 80 mg/dL and/or high-density lipoprotein cholesterol: men < 40 mg/dL, women < 46 mg/dL and/or triglycerides > 150 mg/dL), elevated blood pressure values (SBP > = 140 mmHg and/or DBP > = 90 mmHg), and a family history of premature CVD (men aged < 55 years; women aged < 65 years).

## Results

### Preliminary correlational analyses

All of the correlations among the study variables are presented in Table 2. Following the guidelines presented by Cohen [135], we interpreted correlations as measures of effect size. Specifically, correlations were considered weak (|.10| < r < |.29|), moderate (|.30| < r < |.49|), or strong (|.50| < r < |1|).

**Table 2:** Correlations among study variables.

Variable		2	3	4	5	6	7	8	9	10	11	12	13	14
Personality														
	1. Type A personality	.38***	.12**	.21***	.19***	.03	-.08*	.11**	.04	-.03	.01	.11**	.10**	.10**
	2. Type D—Negative affectivity		.09*	.57***	.46***	-.29***	-.35***	.34***	.22***	-.03	-.08*	-.06	.08*	.03
	3. Type D—Social inhibition			-.01	.13**	-.05	-.12**	.02	-.01	.02	-.08*	-.00	-.02	-.02
Psycho-emotional distress														
	4. Anxiety				.62***	-.32***	-.41***	.49***	.39***	.00	-.05	-.12**	.08*	-.06
	5. Depression					-.40***	-.46***	.46***	.40***	-.05	-.07	-.12**	.08*	.05
Positive orientation														
	6. Self-esteem						.39***	-.27***	-.23***	.06	.08*	.15***	-.04	.00
	7. Optimism							-.33***	-.20***	.06	.11**	.10*	-.03	-.03
Health and illness representations														
	8. Illness Perception								.38***	-.07	-.05	-.06	.16***	.05
	9. Self-rated health									.02	-.10*	-.08*	-.07	-.05
Lifestyle														
	10. Diet										.09*	-.06	-.28***	-.06
	11. Physical activity											.06	-.08*	-.09*
	12. Alcohol consumption												.10*	.12**
	13. Smoking behavior													.23***
Clinical evaluation														
	14. Total Cardiovascular Risk Index													-

* p < .05

** p < .01

*** p < .001

Type A personality was moderately and positively related to negative affectivity and weakly and positively associated with social inhibition, anxiety, depression, illness perception, and alcohol consumption; moreover, Type A personality was related to optimism, smoking behaviour, and to the TCRi, but this association could be disregarded because of a low effect size. Negative affectivity was strongly and positively related to anxiety, moderately and positively associated with depression and illness perception, and moderately and negatively related to optimism. Moreover, negative affectivity was weakly and positively associated with worse self-rated health and weakly and negatively related to self-esteem. Finally, negative affectivity was associated with social inhibition, physical activity, and smoking behaviour, but this association could be disregarded because of a low effect size. Social inhibition was weakly and positively associated with depression and weakly and negatively related to optimism; moreover, social inhibition was related to physical activity, but this association could be disregarded because of a low effect size.

### Cluster analysis on Type A and D personality

A two-step clustering procedure was used to typify patients according to their continuous Type A and D personality levels. This approach used a combination of hierarchical and nonhierarchical clustering methods [136]. Ward’s [137] hierarchical method, followed by the non-hierarchical K-means method, were applied by the software SLEIPNER 2.1 [138]. Before these analyses were performed, missing data from the items were substituted using hot deck imputation [139, 140]. According to Roth [140], this procedure is recommended when the percentage of missing data is lower than 10% regardless of the pattern of the missing data. It should be noted that participants with many missing values (more than 10%) should be excluded from the analyses. Hot deck imputation replaces a missing value with the value of a similar “donor” in the dataset. In our analysis, the “donor” was selected according to the sex and age of the participants. Because the percentage of missing data was very low (0.3%), some values were imputed using hot deck imputation, and one case that had too many missing values was excluded.

**Fig 1:**
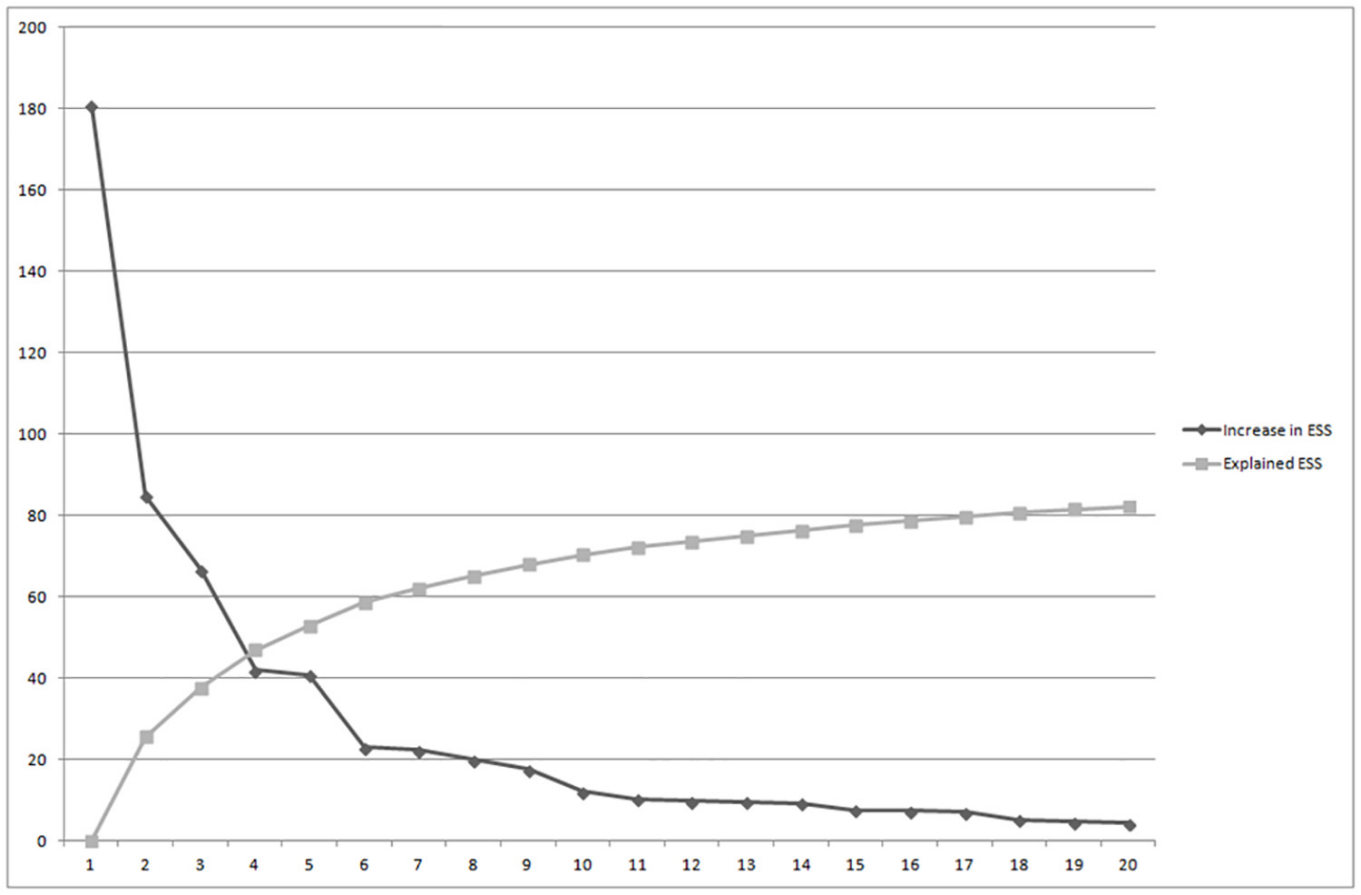
Error sum of square plot for the activity cluster solution. ESS = error sum of squares.

Ward’s hierarchical agglomerative clustering technique starts with observations as separate clusters and then gradually links them together based on their squared Euclidean distance. This algorithm involves a fusion process that joins the cluster by minimizing increases in the within-cluster or error sum of square (ESS) while maximizing the variance between clusters [141, 142]. Regarding the hierarchical method, different solutions were chosen based on the size of the change in the ESS values between adjacent cluster solutions, as suggested by Bergman [143]. A substantial decrease in the ESS indicated that during the partition process, non-homogeneous individuals were confounded in the same cluster. Fig 1 is a scree-type plot that shows the change in the ESS according to the cluster solution. The four- and six-cluster solutions were retained for further analysis.

In Ward’s method, clusters were fused in step one, and the participants remain together in all later steps, resulting in non-optimal solutions. Therefore, each solution was subsequently used as the initial cluster centre for a non-hierarchical K-means clustering procedure in which, in subsequent steps, all of the participants were assigned to the most similar cluster based on their Euclidean distance from the initial cluster centres. Non-hierarchical procedures reduce the total ESS of the cluster solution, produce more homogeneous clusters and further improve the preliminary cluster solution through an iterative process [144]. In this non-hierarchical method, four indices were used to evaluate the optimal number of clusters: the C-index [145], the G (+) index [146], the Gamma index [147], and the point-biserial correlation [148]. The minimum value of the former two indices and the maximum of the latter two suggested the optimal number of clusters to be retained and hence provided the best cluster solution. Moreover, cluster homogeneity coefficients below 1 are considered desirable because lower values indicate greater homogeneity, and higher values indicate reduced homogeneity [149]. Table 3 presents the fit indices of the two solutions, indicating that the six-cluster solution was the most appropriate. In fact, although the point-biserial correlation was higher in the four-cluster solution, the C-, the G(+), and Gamma indices were more appropriate in the six-cluster solution. Moreover, these solutions showed differences in the ESS that were higher in the “larger” solution (63.70) than in the “smaller” one (52.47). In addition, as a further confirmation of this choice, all homogeneity coefficients of the six-cluster solution were below one (cl1 = 0.63, cl2 = 0.79, cl3 = 0.73, cl4 = 0.87, cl5 = 0.72, cl6 = 0.68), indicating that all clusters were reasonably homogenous. Collectively, these considerations identified the six-cluster solution as the optimal one.

**Table 3:** Fit indices of the four- and six-cluster solution identified through K-means cluster analysis.

Goodness-of fit indices	4 cluster	6 cluster
Point-biserial correlation	.35	.33
C-index	.15	.12
Gamma	.56	.66
G (+)	.08	.05
Explained error sum of squares	52	64

**Fig 2:**
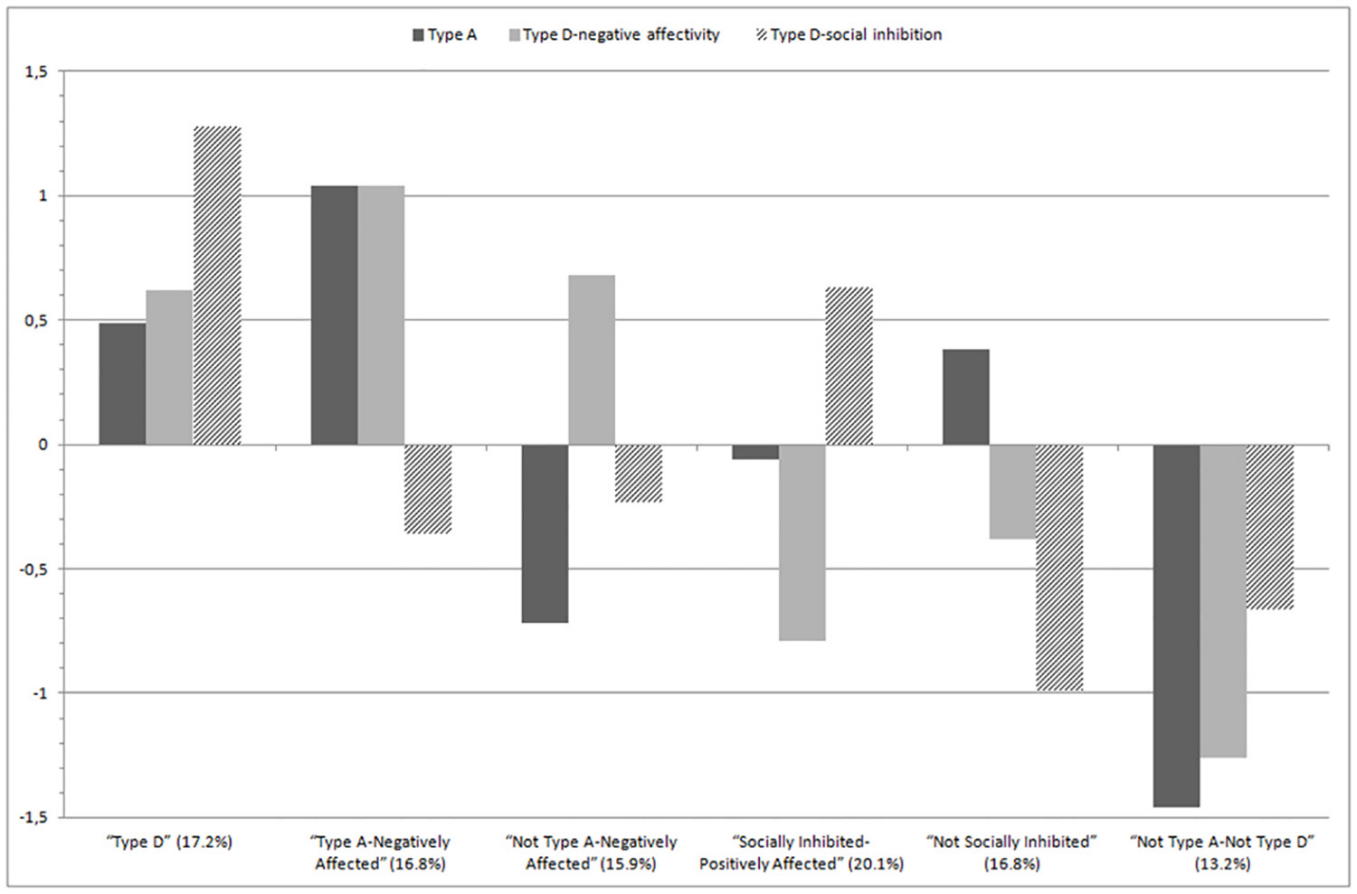
Z-scores for Type A, Type D-negative affectivity, and Type D-social inhibition for the final six-cluster solution.

Fig 2 presents the final cluster solution; the Y-axis represents z-scores. Because the clusters were defined using z-scores for the total sample, each cluster’s mean z-scores indicate the distances between the cluster means and the total sample’s standardized mean [150]. These ranges, expressed in standard deviation units, were interpreted as effect sizes. Analogous to Cohen’s [135] *d*, a 0.2 standard deviation is a small effect, a 0.5 standard deviation is a medium or moderate effect, and a 0.8 standard deviation is a large effect.

Cluster 1, made up of 122 participants (17.2% of the sample), was labelled “Type D” and was characterized by high scores on negative affectivity and social inhibition and moderately high scores on Type A personality characteristics. Cluster 2, made up of 119 participants (16.8% of the sample), was labelled “Type A-Negatively Affected” and was characterized by high scores on Type A personality characteristics and negative affectivity and moderately low scores on social inhibition. Cluster 3, made up of 113 participants (15.9% of the sample), was labelled “Not Type A-Negatively Affected” and was characterized by high scores on negative affectivity and low scores on Type A personality characteristics. Cluster 4, made up of 143 participants (20.1% of the sample), was labelled “Socially Inhibited-Positively Affected” and was characterized by high scores on social inhibition and low scores on negative affectivity. Cluster 5, made up of 119 participants (16.8% of the sample), was labelled “Not Socially Inhibited” and was characterized by low scores on social inhibition, moderately low scores on negative affectivity, and very moderately high scores on Type A personality characteristics. Cluster 6, made up of 94 participants (13.2% of the sample), was labelled “Not Type A-Not Type D” and was characterized by low scores on Type A personality characteristics, negative affectivity, and social inhibition.

We used a double-cross validation process to examine the internal replicability and stability of this cluster solution [151]. According to this procedure, the full sample of participants should be split into two halves, and the two-step clustering procedure should be applied to each half. The two solutions (obtained from the two halves of the sample) should then be compared to determine the agreement between the original clusters and Cohen’s kappa [152], and the two resulting kappa values should be averaged [153, 154]. A mean k-value of at least 0.60 is considered acceptable [155]. The sample was split into two halves depending on the pathology (HYP vs ACS), and the full two-step procedure was applied within each subsample. These new clusters were compared with the original cluster-solution using Cohen’s kappa; the two resulting kappas were averaged. The mean kappa was .78, attesting to the stability of the six-cluster solution. Similarly, no significant differences in the distribution of pathology among the six clusters were found (χ^2^ (5) = 6, *p* = *n*.*s*.), demonstrating that there was no relationship between the composition of the clusters and the diseases investigated. In fact, in the “Type D” cluster, there was 7.2% HYP vs 10.0% ACS patients; in the “Type A-Negatively Affected” group, there was 7.6% HYP vs 9.2% ACS patients; in the “Not Type A-Negatively Affected” cluster, there was 8.7% HYP vs 7.2% ACS patients; in the “Socially Inhibited-Positively Affected” group, there was 10.7% HYP vs 9.4% ACD patients; in the “Not Socially Inhibited” cluster, there was 7.6% HYP vs 9.2% ACS patients; finally, in the “Not Type A-Not Type D” group, there was 6.6% HYP vs 6.6% ACS patients.

### External correlates of the clusters

After cluster identification, a series of tests were performed to compare the external variables of the identified types. Cluster membership was used as an independent variable, and demographic, psychological, lifestyle, and clinical variables were considered dependent variables.

### Demographic and social connectedness indicators

A univariate analysis of variance (ANOVA) showed that the six clusters did not differ with respect to mean age (*F* (5, 709) = 0.67, *p* = n.s., ƞ^2^ = .01). Moreover, as shown in Table 4, the results of the χ^2^ analyses revealed significant differences among the six clusters in terms of gender, employment status, and number of friends. Men were overrepresented in the “Type D”, “Not Socially Inhibited”, and “Socially Inhibited-Positively Affected” groups, while women were more represented in the “Not Type A-Negatively Affected” and “Not Type A-Not Type D” clusters. With respect to employment status, retired individuals were overrepresented in the group identified as “Type A-Negatively Affected”, retired individuals with some work activities were overrepresented in the “Not Socially Inhibited” group, unemployed individuals were overrepresented in the “Not Type A-Negatively Affected” group, and homemakers were overrepresented in the “Not Type A-Not Type D” group. Moreover, the “Type D” cluster was marked by an overrepresentation of participants with 1–5 friends. The “Type A-Negatively Affected” group was characterized by a higher representation of patients with no friends. The “Socially Inhibited-Positively Affected” cluster was marked by an overrepresentation of patients with 6–10 friends. The “Not Socially Inhibited” group was characterized by an overrepresentation of participants with more than 10 friends. No differences were found with respect to marital status (χ^2^ (5) = 21, *p* = *n*.*s*.) or educational level (χ^2^ (5) = 26, *p* = *n*.*s*.).

**Table 4:** Significant χ^2^ analyses for the final six-cluster solution for gender, employment status, number of friends, diet and physical activity: percentages and adjusted residuals.

Variable	Clusters						χ^2^
	TD	TANA	NA	SIPA	NSI	NOTAD	
	% (Adj)	% (Adj)	% (Adj)	% (Adj)	% (Adj)	% (Adj)	
Gender							57.18***
Male	13.5 (2.1)	12.4 (0.9)	7.3 (-6.3)	16.6 (3.5)	13.1 (2.0)	7.7 (-2.8)	
Female	3.7 (-2.1)	4.4 (-0.9)	8.6 (6.3)	3.5 (-3.5)	3.7 (-2.0)	5.5 (2.8)	
Employment status							62.85***
Employed	9.9 (0.6)	8.1 (-1.7)	8.6 (-0.2)	11.9 (1.1)	10.2 (1.4)	6.4 (-1.4)	
Retired	1.3 (0.5)	1.8 (2.2)	0.8 (-0.5)	0.7 (-1.6)	1.1 (0.2)	0.6 (-0.9)	
Retired with some work activities	0.7 (-1.1)	1.0 (-0.2)	0.4 (-1.8)	1.6 (0.8)	1.8 (2.3)	0.8 (0.0)	
Unemployed	0.3 (-1.8)	0.4 (-1.3)	2.5 (6.0)	0.4 (-1.7)	0.6 (-0.8)	0.6 (-0.2)	
Homemaker	5.1 (0.6)	5.5 (1.4)	3.5 (-1.4)	5.5 (0.0)	3.0 (-2.6)	4.8 (2.1)	
Number of friends							44.14***
None	0.4 (-0.6)	1.1 (2.2)	0.3 (-1.0)	0.4 (-0.9)	0.8 (1.1)	0.3 (-0.7)	
1–5 friends	12.4 (4.2)	9.5 (0.4)	9.2 (0.6)	9.3 (-2.2)	7.6 (-2.2)	6.8 (-0.8)	
6–10 friends	2.4 (-1.6)	2.4 (-1.5)	3.2 (0.3)	5.6 (3.0)	2.4 (-1.5)	3.1 (1.1)	
More than 10 friends	2.0 (-3.2)	3.8 (0.0)	3.2 (-0.6)	4.7 (0.2)	5.8 (3.5)	3.1 (0.2)	
Diet							25.58*
Inadequate	4.9 (2.2)	3.1 (-0.8)	4.4 (1.7)	2.8 (-2.4)	3.4 (-0.3)	2.7 (-0.3)	
Partial	6.5 (-2.7)	9.2 (1.4)	7.2 (-0.8)	10.5 (-0.9)	9.3 (1.7)	6.1 (-0.6)	
Good	5.1 (0.5)	4.0 (-1.1)	4.2 (-0.3)	6.4 (1.1)	3.7 (-1.5)	4.5 (1.4)	
Excellent	0.7 (1.7)	0.6 (1.0)	0.1 (-1.0)	0.4 (0.0)	0.3 (-0.4)	0.0 (-1.5)	
Physical activity							18.68**
Inactive	16.1 (1.1)	16.1 (2.3)	15.1 (1.5)	17.5 (-1.7)	13.9 (-3.3)	12.2 (0.2)	
Active	1.1 (-1.1)	0.6 (-2.3)	0.8 (-1.5)	2.5 (1.7)	2.8 (3.3)	1.1 (-0.2)	

Note: TD = “Type D”, TANA = “Type A-Negatively Affected”, NA = “Not Type A-Negatively affected”, SIPA = “Socially Inhibited-Positively Affected”, NSI = “Not Socially Inhibited”, NOTAD = “Not Type A-Not Type D”, % = percentages computed on total, Adj = adjusted residuals.

* p < .05

** p < .01

*** p < .001

### Psychological variables

Table 5 presents the means and standard deviations for the indicators of anxiety, depression, self-esteem, optimism, illness perception, and self-rated health. Table 5 also shows the results of the Levene’s test to check for the homogeneity of the variances for each dependent variables. If Levene’s test is significant indicates that the homogeneity of variances was violated; in this case, Welch's robust test was used to compare the clusters, followed by the Tamhane post-hoc tests for posteriori tests. If Levene’s test is not significant indicates that homogeneity of variances was respected; in this case, a series of one-way ANOVA was used to compare the clusters, followed by the Tukey post-hoc test. The comparison and the post hoc tests revealed significant differences for all variables.

**Table 5:** Means and standard deviation for psycho-emotional distress, positive orientation, health and illness representations, lifestyle (Alcohol consumption and Smoking behavior), and clinical evaluation (Total Cardiovascular Risk Index) for the final six-cluster solution. Results from comparisons and post-hoc tests.

Variable (range)	Clusters						Levene’s test	F/Welch test value	ƞ^2^
	TD	TANA	NA	SIPA	NSI	NOTAD	(5, 704)	(gdl)	
	Mean (SD)	Mean (SD)	Mean (SD)	Mean (SD)	Mean (SD)	Mean (SD)			
Psycho-emotional distress									
Anxiety (0–21)	8.27**b**	9.87**a**	8.86**a b**	5.15**c d**	6.05**c**	4.54**d**	7.98***	52.58***	0.26
	(3.87)	(3.53)	(3.97)	(2.50)	(3.30)	(2.73)		(5, 318.20)	
Depression (0–21)	6.17**a**	6.29**a**	5.73**a**	3.70**b**	3.52**b**	2.96**b**	6.68***	28.59***	0.16
	(3.60)	(3.23)	(3.13)	(2.84)	(2.72)	(2.45)		(5, 322.63)	
Positive orientation									
Self-esteem (1–4)	3.16**c**	3.17**b c**	3.12**c**	3.41**a**	3.43**a**	3.36**a b**	3.54**	9.90***	0.06
	(0.52)	(0.61)	(0.50)	(0.41)	(0.49)	(0.42)		(5, 320.43)	
Optimism (1–5)	3.33**b**	3.39**b**	3.37**b**	3.81**a**	3.89**a**	3.95**a**	3.27**	17.76***	0.11
	(0.72)	(0.85)	(0.88)	(0.67)	(0.58)	(0.68)		(5, 319.84)	
Health and illness representations									
Illness perception (8–40)	22.64**a**	23.39**a**	22.07**a**	19.78**b**	20.34**b**	19.68**b**	0.62	14.73***	0.09
	(4.58)	(4.81)	(4.27)	(4.25)	(4.67)	(4.36)		(5, 709)	
Self-rated health (1–5)	2.52**a b c**	2.70**a**	2.58**a b**	2.31**c**	2.31**c**	2.34**b c**	1.32	6.38***	0.04
	(0.72)	(0.67)	(0.62)	(0.73)	(0.80)	(0.70)		(5, 709)	
Lifestyle									
Alcohol consumption (1–4)	1.57**a b c**	1.70**a b**	1.30**c**	1.75**a**	1.77**a**	1.39**b c**	2.63*	6.12***	0.04
	(0.86)	(0.77)	(0.89)	(0.72)	(0.90)	(0.84)		(5, 317.86)	
Smoking behavior (0–4)	0.98	1.03	0.64	0.79	0.97	0.65	4.57***	2.25*	0.01
	(1.30)	(1.37)	(1.06)	(1.28)	(1.33)	(1.09)		(5, 321.85)	
Clinical evaluation									
Total Cardiovascular Risk Index (0–9)	4.58	5.02**a**	4.20**b**	4.39	4.61	4.41	1.27	2.34*	0.02
	(1.90)	(1.86)	(1.99)	(1.95)	(2.04)	(2.09)		(5, 709)	

Note: TD = “Type D”, TANA = “Type A-Negatively Affected”, NA = “Not Type A-Negatively affected”, SIPA = “Socially Inhibited-Positively Affected”, NSI = “Not Socially Inhibited”, NOTAD = “Not Type A-Not Type D”. Different letters indicate significant differences among clusters. Where Levene’s test was significant, Welch's robust test was used to compare the clusters, followed by Tamhane post-hoc test; where Levene’s test was not significant, univariate analysis of variance test was used to compare the clusters, followed by Tukey post-hoc test.

* p < .05

** p < .01

*** p < .001

Regarding anxiety, the “Not Type A-Not Type D” cluster scored lowest, and the “Type A-Negatively Affected” and “Not Type A-Negatively Affected” clusters scored highest. Regarding depression and illness perceptions, those two clusters and the “Type D” cluster scored higher than the other three clusters. Self-esteem and optimism were higher for the “Socially Inhibited-Positively Affected”, “Not Socially Inhibited”, and “Not Type A-Not Type D” clusters than for the other three. Regarding self-rated health, the “Socially Inhibited-Positively Affected” and “Not Socially Inhibited” clusters reported better health status than the “Type A-Negatively Affected” cluster.

### Lifestyle

The results of the χ^2^ analyses (Table 4) revealed significant differences among the six clusters in terms of diet and physical activity. The “Type D” cluster was characterized by an overrepresentation of patients with an inadequate diet, whereas the “Socially Inhibited-Positively Affected” cluster was marked by an underrepresentation of patients with an inadequate diet. The “Not Socially Inhibited” cluster was marked by an overrepresentation of patients who met the target for healthy physical activity, whereas the “Type A-Negatively Affected” cluster was characterized by an overrepresentation of patients who were physically inactive.

These six clusters differed with respect to alcohol intake, as indicated by a Welch's robust test (Table 5). The “Socially Inhibited-Positively Affected” and “Not Socially Inhibited” clusters reported higher alcohol consumption than the “Not Type A-Not Type D” and “Not Type A-Negatively Affected” clusters.

These six clusters showed significant differences with respect to cigarette smoking behaviour, as indicated by a Welch's robust test, but the Tamhane post-hoc tests did not show significant differences among cluster for this dependent variable. Anyway, the “Type A-Negatively Affected” cluster tended to smoke more than the “Not Type A-Not Type D” and “Not Type A-Negatively Affected” clusters.

### Clinical evaluation

The one-way ANOVA results shown in Table 5 revealed differences among the six clusters on the TCRi. As indicated by Tukey post hoc comparisons, the “Type A-Negatively Affected” group had a greater presence of CVD risk factors than the “Not Type A-Negatively Affected” cluster.

## Discussion

The present study aimed to identify how Type A and Type D personalities might integrate to form distinct and meaningful profiles or clusters in two groups of patients: those affected by ACS, the most common type of CVD, and those with essential HYP, the main risk factor for CVD. Type A and Type D are the personality profiles most often associated with the prognosis of CVD [2–4, 155], but previous studies have focused on these personality types separately.

Six clusters were identified, and each was characterized by its own unique and specific profile of the Type A and D personality combination. First, the Type D cluster, characterized by high scores for social inhibition and negative affectivity, resembled the typology originally identified as distressed personality [3, 4, 52, 56, 58]. This cluster was also characterized by moderately high scores on Type A personality characteristics. It is possible that a moderate presence of Type A characteristics within Type D personality could be typical of this profile, as suggested by Perbandt et al. [67], but this finding should be investigated more widely in future research that considers the combination of these personality types. Second, the Type A-Negatively Affected typology somewhat resembled previous clusters and has been characterized by high levels of Type A in other populations [1, 2, 19, 156]; however, in the present study, this group also had a high score on one aspect of Type D personality: negative affectivity. Therefore, this group was characterized by high scores for Type A personality and negative affectivity. Third, two groups were characterized by high scores for one of the two distinct dimensions of Type D personality: negative affectivity and social inhibition. The Not Type A-Negatively Affected cluster was characterized by high scores for negative affectivity but low scores for Type A personality. In contrast, the Socially Inhibited-Positively Affected group was characterized by high scores for social inhibition but low scores for negative affectivity, the other characteristic of Type D personality. Fourth, the last two clusters were characterized by the absence of detrimental characteristics related to Type A and D personality, and they represent positive profiles. The Not Type A-Not Type D cluster was characterized by low scores for both Type A and Type D personality characteristics, whereas the Not Socially Inhibited group, despite moderately high scores for Type A personality characteristics, scored low for social inhibition and moderately low for negative affectivity.

Moreover, in line with our hypotheses, we found a cluster characterized by high levels of Type D characteristics, a cluster marked by the presence of Type A and Type D characteristics together, and clusters with features that were the reverse of these. In contrast with our hypotheses, we did not find any cluster marked by high levels of Type A personality alone; instead, the characteristics of competitiveness, impulsivity, job involvement, leadership, and hostility were combined with Type D personality characteristics. Moreover, our results demonstrated the independence between the clusters and the diseases investigated (HYP and ACS), allowing us to establish the internal replicability and stability of the six-cluster solution for the two pathologies. This finding represents an additional novel element because previous studies have primarily focused on a homogenous type of disease, such as acute illnesses. Given that HYP is a major risk factor for other CVDs, the study of HYP patients and their similarity to ACS patients is certainly relevant for investigating and clarifying the pathways between psychological factors and CVD and for making suggestions regarding the role of these factors in the pathogenesis of these diseases.

Moreover, a double-cross validation process showed that these six groups were internally stable and replicable. Furthermore, as hypothesized, despite a weak pattern of associations (except for the correlations between Negative Affectivity and psycho-emotional distress factors) among the Type A and Type D personality variables and other components considered in the study, the six groups were externally validated in terms of demographic, psychological, lifestyle, and clinical indicators. As expected, the clusters with high levels of Type A and/or Type D characteristics showed a less-positive profile of external correlates compared with groups with more optimal profiles. The Type A-Negatively Affected cluster (i.e., the group with a combination of Type A and Type D characteristics) was the most negative profile identified. This group was characterized by an overrepresentation of retired patients with no friends. Moreover, these patients presented high levels of anxiety and depression, a more negative illness perception, and low levels of self-rated health. Regarding lifestyle and clinical indicators, this group was characterized by an overrepresentation of patients with unhealthy levels of physical activity and a greater presence of CVD risk factors. The results related to anxiety and depression might seem inconsistent with previous studies that found weak associations among these factors and Type A characteristics in CVD populations [157–159]. However, other research has found that negative affect is associated with increased anxiety and depression in patients with chronic disease [93, 160] and with more physical symptoms, a negative health perception and lower levels of self-rated health [93–95]. Therefore, our results seem to combine findings from previous research investigating different characteristics related to CVD, suggesting that the combination of Type A personality and negative affectivity could have a major impact on the psychological, social, and physical functioning of CVD patients. Consistent with our results, previous findings have demonstrated that Type A individuals showed hyperlipidaemia (higher average levels of serum cholesterol and triglyceride) and higher blood pressure compared with Type B subjects [161, 162]. Regarding physical activity, our results seem to confirm previous research that found an association between Type A personality and physical inactivity [91, 163].

Additionally, the Type D and Not Type A-Negatively Affected clusters were characterized by a rather negative profile of the external variables. In the Type D cluster, the patients were primarily men with few friends. They presented high scores for anxiety and depression, a negative illness perception and low levels of self-rated health, self-esteem, and optimism. Moreover, this cluster was characterized by unhealthy dietary habits. These results are consistent with previous findings demonstrating that Type D personality was correlated with anxiety and depression [48, 96], poor health-related quality of life [97, 98], more negative illness perceptions [58, 99], and low positive orientation [56]. Moreover, Type D personality was related to unhealthy behaviours, such as a poor diet and a higher intake of fat, smoking, and poor treatment adherence [100, 101]. In the Not Type A-Negatively Affected group, there was an overrepresentation of women and unemployed patients. These patients were also characterized by high scores for anxiety, depression, and illness perception and low scores for perceived health, self-esteem, and optimism. However, along with these lifestyle and clinical indicators, these patients also reported lower alcohol consumption than other clusters and a reduced presence of CVD risk factors. These findings might be related to the very high participation of women; according to the literature, women tend to drink less alcohol than men [164, 165] and to have lower CVD rates than men until menopause, probably because the female sex hormone acts on white blood cells to inhibit them from adhering to the insides of blood vessels [166, 167]. Additionally, the higher scores for psycho-emotional distress and the low scores for positive orientation and health and illness representations could also be influenced by the overrepresentation of women: research on sex-linked stereotypes supports the belief that women are more emotional than men [168–170].

The Socially Inhibited-Positively Affected, Not Socially Inhibited, and Not Type A-Not Type D groups were characterized by more positive profiles. The patients in the Socially Inhibited-Positively Affected cluster were mainly men who scored low on anxiety, depression, and negative illness perception but had high self-esteem, optimism, and self-rated health. Moreover, this cluster was characterized by an underrepresentation of patients with an inadequate diet and by a high reported alcohol consumption; such high alcohol consumption patterns could be related to the richer social life that these patients declare that they have. The Not Socially Inhibited group was mainly composed of men who were retired but still working and who had numerous friends. This group was also characterized by low levels of depression and negative illness perceptions and high self-esteem, optimism, and self-rated health. Furthermore, this cluster was characterized by an overrepresentation of patients with high alcohol consumption, but these patients met the target for healthy physical activity. Finally, the Not Type A-Not Type D group was characterized by an overrepresentation of women who were homemakers and had low scores for anxiety, depression, and negative illness perception and high levels of self-esteem, optimism, and self-rated health. These results are in line with previous studies demonstrating that people characterized as Type B—namely, those without Type A characteristics—or non-Type D showed lower psycho-emotional distress, higher positive orientation, better health and illness representations, and better health-related behaviours than Type A or Type D individuals [54, 58, 92, 161].

In sum, our cluster-analysis findings emphasize the need to examine combinations of personality typologies rather than focusing on a single typology; in fact, we found clusters marked by Type D characteristics, by the presence of Type A and Type D characteristics together, and by reverse features, but we did not find any cluster marked by high levels of Type A characteristics only. The combination of these personality patterns seems to have important clinical implications.

Despite its strengths, our study also has some limitations. First, cluster analysis is an empirically driven approach and assumes the perfect classification of observations into clusters. Consequently, the outcomes are conditioned by sample characteristics, which could pose a problem for the subsequent ANOVAs and chi-square tests. Further studies are needed to confirm our typologies: the clusters need to be replicated in independent samples of individuals with HYP and ACS. Despite the stability of the typologies identified for the two different pathologies, it would also be interesting to examine data from samples of patients with various CVDs (e.g., heart failure, chronic ischaemic heart disease, percutaneous coronary intervention and coronary artery bypass surgery for reduced coronary reserve) to identify whether Type A and Type D combined personality represents an important integration of characteristics that has clinically meaningful implications for other CVDs. Second, an important limitation of the present study is its cross-sectional design. Longitudinal research is necessary to determine the stability of these clusters over time; in fact, we expect that Type A and Type D personality and several of the external variables assessed mutually reinforce each other over time. Moreover, an examination of the predictive value of the psychological profiles detected in this study could be very promising in terms of adding new insights to current knowledge of the association between psychological factors and CVD. Such longitudinal studies would also allow an investigation of the degree to which these clusters interact or transact over time with illness-specific tasks, such as changing lifestyle habits and controlling modifiable risk factors, which are cardinal aspects of CVD prevention and treatment. Moreover, these longitudinal data could also be concentrated to analyse personality data in terms of the interactions of continuous variables because various researchers have raised issues about the validity of conceptualizing personality types as categorical types [66]. Third, additional studies are necessary to assess whether various interventions yield different outcomes for different personality profiles to improve individuals’ self-management of their health condition. Fourth, additional studies may consider how the Type A and D personality factors could combine with other variables, also using positive and less investigated aspects, such as positive expectations related to the diseases since a previous study has found that, independently by disease severity, depressive symptoms, and social isolation, CHD patients with high levels of expectation of being able to recover had a 21% reduction in cardiac mortality over a 15 year follow-up period [171]. Finally, a more ecologically valid method would be useful to assess the truthfulness of our participants’ reported information. For instance, an experience sampling method that assesses diet, physical activity, alcohol consumption, and smoking could be useful in validating the clusters with an online measure of behaviour. Similarly, although self-report questionnaires are considered a good method for assessing different psychological variables, structured interviews are the gold standard, and they should be used to gather more detailed information regarding various factors in CVD patients, such as Type A behaviour patterns and anxiety and depression; interviews could also be used to make clinical diagnoses regarding these variables, and this information could be used to validate the clusters.

In conclusion, the present study emphasized the need to examine types of individuals through cluster analysis in CVD research and clinical practice. This holistic and person-centred approach allows the study of individual functioning through an integrative view of the person, one that cannot be completely understood with traditional, more variable-centred approaches that consider individual components of the person in isolation [68, 69]. Cluster differences were based on clinically relevant variables such as psycho-emotional distress, positive orientation, health and illness representations, lifestyle, and clinical indicators. The findings of this study should encourage researchers and practitioners to focus their attention on combinations of personality typologies that provide highly relevant information for clinical interventions, including both the primary and secondary prevention of CVD. A typological approach could be useful to screen at-risk patients who do not meet the target of healthy behaviour. An ordinary personality screening of HYP and ACS patients could provide valuable information for differentiating and tailoring health-education programmes to include specific tasks; for example, education and intervention could focus on a specific unhealthy lifestyle behaviour or a modifiable risk factor instead of giving generic and undifferentiated information to all patients. Therefore, the present study could set the stage for an integrative person-centred approach for patients with CVD with a focus on identifying meaningful subgroups, and it could consequently provide a framework for customizing treatment for specific groups of patients to foster individuals’ self-management of their health condition [172].

## Supporting Information

S1 Dataset Dataset of the study. (SAV)
